# The T-MSIS Analytic Files (TAF) Analysis Reporting Checklist: A Guide for Research Using Medicaid Claims Data

**DOI:** 10.1001/jamahealthforum.2025.3622

**Published:** 2025-10-03

**Authors:** William L. Schpero, K. John McConnell, Greta Bushnell, Alina Denham, Patience M. Dow, Shashi N. Kapadia, Stephan R. Lindner, Hillary Samples, Lindsay Shea, Kelsey Watson, Sarah H. Gordon

**Affiliations:** Department of Population Health Sciences, Weill Cornell Medical College, New York, New York (Schpero, Kapadia); Cornell Health Policy Center, Cornell University, New York, New York (Schpero, Kapadia); Cornell Center for Health Equity, Cornell University, New York, New York (Schpero); Department of Emergency Medicine, Oregon Health & Science University, Portland (McConnell, Lindner); Center for Health Systems Effectiveness, Oregon Health & Science University, Portland (McConnell, Lindner, Watson); Department of Biostatistics and Epidemiology, Rutgers School of Public Health, Piscataway, New Jersey (Bushnell); Center for Pharmacoepidemiology and Treatment Science, Rutgers Institute for Health, Health Care Policy and Aging Research, New Brunswick, New Jersey (Bushnell, Samples); Department of Family, Population, and Preventive Medicine, Renaissance School of Medicine at Stony Brook University, Stony Brook, New York (Denham); Department of Health Services, Policy, and Practice, Brown University School of Public Health, Providence, Rhode Island (Dow); Division of Infectious Diseases, Weill Cornell Medical College, New York, New York (Kapadia); Department of Health Behavior, Society and Policy, Rutgers School of Public Health, Piscataway, New Jersey (Samples); Department of Health Management and Policy, Drexel University Dornsife School of Public Health, Philadelphia, Pennsylvania (Shea); Department of Health Law, Policy, and Management, Boston University School of Public Health, Boston, Massachusetts (Gordon)

## Abstract

**IMPORTANCE:**

Medicaid is the single largest source of health care coverage in the US, but health policy research on the Medicaid program has historically lagged research on Medicare due to limited availability of high-quality administrative claims data across states. In 2019, the US Centers for Medicare & Medicaid Services released the T-MSIS Analytic Files (TAF), a new-generation federal Medicaid claims dataset that has catalyzed policy-relevant research on the Medicaid program. TAF data are highly complex, however, with meaningful differences in quality across states, years, and data elements. There is an urgent need for standardized reporting guidelines to ensure TAF-based research is high quality and reproducible.

**OBJECTIVE:**

To develop a checklist to guide reporting of research using the TAF data.

**EVIDENCE REVIEW:**

The development of the TAF Analysis Reporting Checklist was led by a subcommittee of the Medicaid Data Learning Network (MDLN), a national consortium of research teams focused on developing and disseminating best practices for conducting health services research with the TAF data. The subcommittee first created a draft checklist drawing from published technical guidance on proper use of the TAF data, as well as published analyses of TAF data quality. This draft was iteratively refined based on feedback from (1) MDLN members; (2) the MDLN Advisory Group, composed of leaders in academia, government, and industry with Medicaid claims experience; (3) editors of health policy journals; and (4) the broader Medicaid research community.

**FINDINGS:**

The final checklist includes 4 categories of items that are recommended for reporting in studies using the TAF data. This includes (1) details on the specific data used (files, years, release versions, and size of the data extract), (2) how the analytic sample was defined (eligibility criteria, enrollment span, and scope of benefits), (3) what states and/or territories were excluded from the analysis based on data quality concerns (and the exclusion criteria used to do so), and (4) additional information on special considerations, including use of spending data and changes in data quality over time.

**CONCLUSIONS AND RELEVANCE:**

The TAF Analysis Reporting Checklist represents a consensus effort to identify items researchers should report to promote transparency and reproducibility in TAF-based studies. This reporting is a key step in safeguarding the quality of research used to inform Medicaid policy.

## Introduction

Medicaid, the public health insurance program for low-income populations, now provides health care coverage for more than 78 million people—almost 1 in 4 individuals residing in the US. Medicaid covers more than one-third of children, more than 40% of all births, and is the primary source of coverage for almost 60% of people residing in nursing homes.^1–3^ Medicaid spending exceeded $800 billion in 2022, representing 18% of national health expenditures.^4^ For decades, however, research on Medicaid has lagged behind research on Medicare, limiting opportunities to improve program efficiency, evidence-based policymaking, accountability, and transparency for what has become the single largest source of health care coverage in the US.

Barriers accessing high-quality and timely Medicaid data have contributed to limited claims-based Medicaid research relative to Medicare, despite its importance for policy. Medicare claims data—covering the entirety of enrollment, utilization, and spending in the traditional fee-for-service program nationally—have been available for research use for decades. In contrast, high-quality national Medicaid claims data have historically not been available from the federal government. Traditionally, researchers wishing to study Medicaid using administrative data have relied on state-specific claims data or the federal Medicaid Analytic eXtract (MAX) files made available by the US Centers for Medicare & Medicaid Services (CMS). While both data sources have supported important research, they each face limitations: state-specific claims data can be difficult to obtain, requiring state-specific partnerships, and may not produce research generalizable to other states; in turn, the MAX files have significant data quality challenges, have not included comprehensive managed care records, and have only been available with a multiyear lag due to significant processing requirements from state submission to federal release.

In 2019, CMS released the T-MSIS Analytic Files (TAF), the new-generation federal Medicaid claims dataset that replaced the MAX files. The TAF represent a significant improvement in quality over the MAX files and promise to better support timely policy-relevant and clinically relevant research. Yet Medicaid is a program run by states in partnership with the federal government, which leaves room for variation in data collection and reporting practices across states and years, leading to variable data quality and posing challenges for longitudinal and multistate research. As a result, TAF users need to make informed decisions regarding the selection of states with sufficient quality for relevant data elements as well as the benchmarking criteria they use for data quality assessments. There is significant need for structured guidance on how to address and report on variations in data quality for those who generate and consume TAF-based research to ensure the research is high quality, credible, and interpreted and applied appropriately.

This article describes the development and contents of the TAF Analysis Reporting Checklist—a guide for conducting and reporting research using TAF data. Studies have found that reporting guidelines can meaningfully improve the quality of research transparency for both randomized clinical trials (the Consolidated Standards of Reporting Trials [CONSORT] reporting guideline)^5^ and observational studies (the Strengthening the Reporting of Observational Studies in Epidemiology [STROBE] reporting guideline).^6^ Here, we describe the methods we used to develop the TAF Analysis Reporting Checklist, discuss the motivation for including the checklist items, and provide examples of how those items should be addressed in practice. The TAF Analysis Reporting Checklist is intended to be a resource for researchers using TAF as well as the editors, peer reviewers, and readers who assess and consume their work. The goal is to promote transparency and reproducibility in research using TAF data and safeguard the quality of research used to inform Medicaid policy.

## Methods

The TAF Analysis Reporting Checklist development process followed the Guidance for Developers of Health Research Reporting Guidelines published by the Enhancing the Quality and Transparency of Health Research (EQUATOR) Network.^7^

### Development of a Draft Checklist

We formed a subcommittee of the Medicaid Data Learning Network (MDLN) to oversee the development of the checklist. The MDLN, an initiative of AcademyHealth funded by the Commonwealth Fund and Robert Wood Johnson Foundation, is a consortium of more than 70 academic and nonprofit institutions focused on developing and disseminating best practices for conducting policy research with the TAF data. This subcommittee, composed of 11 researchers who regularly produce and review TAF-derived research, first identified the need for structured guidance specific to studies using TAF data. This decision was reached in the context of the increasing use of TAF data in health research and the potential for researchers to inadvertently generate inaccurate or misleading findings due to the unique data quality challenges inherent in the dataset. The subcommittee reviewed the literature for reporting checklists and guidelines with relevance to studies using TAF data, including the STROBE reporting guideline^8^ and the Reporting of Studies Conducted Using Observational Routinely-Collected Data (RECORD) reporting guideline.^9^ The MDLN subcommittee also reviewed the peer-reviewed literature and searched online for technical guidance on use of both the MAX files and TAF. The subcommittee developed a draft checklist informed by existing research guides but tailored to TAF data.

### Feedback and Checklist Revision

The subcommittee presented the draft checklist to the full MDLN membership to solicit feedback. This presentation generated a detailed discussion of each checklist item, including the rationale for inclusion. The subcommittee subsequently revised the checklist and presented the revised version for additional feedback to the MDLN Advisory Group, composed of senior leaders in academia, government, and industry with experience conducting research using Medicaid claims. Following another round of revision, the subcommittee sought input from 3 health policy journal editors; 2 editors provided feedback, and again, the checklist was revised. Finally, the subcommittee presented the checklist in a virtual public webinar for members of the broader Medicaid research community, which also generated a discussion of each checklist item, and was followed by an opportunity to provide written feedback. Stakeholders provided feedback on all aspects of the draft checklist, including its scope (eg, addressing potential overlap with the STROBE checklist), structure (eg, the order in which checklist items should be addressed), and the wording of checklist items (eg, addressing ambiguity in terminology or TAF variable names). The subcommittee incorporated this feedback and finalized the checklist.

## Results

The TAF Analysis Reporting Checklist includes 4 categories of items that should be reported in studies using the TAF data. We include the full checklist with categories, descriptions of each item, and examples of reporting language in the Table. Studies may report checklist items in the main manuscript or an appendix file; the eTable in the Supplement has a version of the checklist to include in study appendices.

### Files, Years, Release Versions, and Data Extract

TAF data include multiple files, including a Demographic and Eligibility File, Inpatient File, Other Services File, Long-Term Care File, and Pharmacy File, as well as supplemental files with additional characteristics for Medicaid-participating facilities and professionals as well as managed care plans. CMS also makes multiple releases of each file available, including preliminary versions that do not reflect full claims runout. To ensure TAF-based research is fully reproducible, researchers should report which years, files, and release versions were used in the construction of their analytic sample. This step is particularly important given state-level variation; different services may be reported in different files across states, and the same service type may not be confined to a single file type. For example, states may vary in the proportion of inpatient claims that are included in the Inpatient File vs the Other Services File.^10^ Lastly, researchers should make clear whether their analytic sample was derived from 100% TAF data or a smaller prespecified data extract (and the specific criteria used to generate any extract used).

### Analytic Sample

Research on the Medicaid population requires clear articulation of the analytic sample, particularly given the wide range of populations covered by the program. If applicable, researchers should provide details on the specific eligibility category codes used to select their study sample. They should also include a description of any additional variables used in conjunction with these codes, such as age or the receipt of specific medical services. Many studies may be limited to specific clinical populations identified by diagnoses or treatments; these should include documentation of diagnosis codes, procedure codes, or medication codes used to generate inclusion and exclusion criteria.

The enrollment span, defined as the minimum period of enrollment required for inclusion in the study sample, must also be clearly specified. Given the frequent churn in and out of the Medicaid program documented by prior studies,^11,12^ it is critical that researchers clarify whether their analysis demands a continuous enrollment period (eg, at least 6 months of continuous enrollment) or if enrollment periods within a broader timeframe are used (eg, a minimum of 12 months during a 3-year study period without requiring consecutive months). This distinction is essential for aligning the study’s methodological approach with its objectives, ensuring that the enrollment criteria are adequately tailored to the research question.

The scope of benefits under Medicaid enrollment is another critical element to report. Researchers should indicate whether their analysis encompasses enrollees with full scope, comprehensive, or restricted benefits.^13^ This step is important to ensure that enrollees under study have access to the relevant range of services; for example, enrollees without full-scope or comprehensive benefits may not be eligible for the Medicaid nonemergency medical transportation benefit and thus studies of the transportation benefit should exclude enrollees with limited or restricted benefits.^14^

More than 75% of Medicaid enrollees are now enrolled in comprehensive managed care plans.^15^ TAF data include encounter records for managed care enrollees, but there can be meaningful differences in data quality for fee-for-service and managed care cohorts.^16,17^ As a result, researchers should be explicit in reporting whether their analysis excluded either fee-for-service or managed care enrollees and the criteria used to do so (eg, enrollment in a comprehensive managed care plan for at least 6 months in the year). Additionally, where relevant, the definition of managed care plan should be explicit, as there are separate variables for plan identification number and plan name (a plan can have multiple identification numbers). Relatedly, researchers should clarify what types of claims records were used in the analysis; in addition to both fee-for-service and managed care encounter records, the claims data include nonencounter records, like capitation payments from states to plans, as well as supplemental payments.

Finally, researchers should clarify whether and how individuals enrolled in both Medicare and Medicaid were included or excluded from the study sample. In general, studies that include dually eligible enrollees but do not use linked Medicare-Medicaid data may be flawed because researchers will not observe the full range of services used by enrollees in the Medicaid data alone. Researchers should describe the criteria used to define dual eligibility, including any operational definitions that guided the exclusion or inclusion of individuals with dual eligibility status in their analysis.

### State and Territory Exclusions

Given the wide variation in Medicaid policy, financing, administration, and data quality across states, it is critical that researchers provide a list of which states and territories were included in any Medicaid analyses using TAF data and explain the criteria guiding any exclusions. In some cases, state inclusion criteria may be determined by the research question, such as restricting to states that implemented a policy of interest during a given time window. State inclusion criteria may also take into account how the state policy context affects the documentation of services in claims; for example, some services provided under a bundled payment may not appear as individual observations in the data. However, in all cases, state inclusion criteria should, to the extent possible, incorporate state-specific and year-specific data quality considerations to increase confidence that similarities or differences across states are driven by the phenomena of interest rather than underlying differences in data quality or completeness.

In developing inclusion criteria based on state-level data quality, researchers should consider both key outcomes of their study and other data elements relevant to the study design, such as elements that may determine selection into the study sample or exposure assignment. For example, a study that examines prescription drug utilization as a primary outcome could exclude states based on the quality of prescription drug claims, along with exclusions based on assessments of TAF enrollment numbers (whether the claims data reflect the correct number of enrollees in a given state and year). However, if that study also relies on diagnosis codes on inpatient claims to identify its cohort, the study should implement state-level exclusions based on inpatient claims data quality indicators as well. Researchers engaged in longitudinal research should note that data quality in TAF varies over time. For longitudinal studies, researchers should be explicit about which states contributed to the analysis in each year.

Benchmarks for data quality can be obtained from a variety of sources. One valuable resource is the Data Quality (DQ) Atlas, an interactive, web-based tool developed specifically for TAF that provides detailed state-by-year data quality indicators across many measures, including enrollment, enrollee demographic characteristics, claims volume, and spending.^18^ For each data attribute, state, year, and release version, the DQ Atlas classifies quality into 5 mutually exclusive categories: low concern, medium concern, high concern, unusable, and unclassified. Researchers should document whether the DQ Atlas was used in making state-level data quality exclusions, and if so, what measures were used and what level of concern was deemed allowable for inclusion of a given state. Studies may rely on multiple DQ Atlas measures in conjunction with other exclusions. An example might be a study that excluded any state identified as high concern or unusable in the DQ Atlas for total Medicaid and CHIP enrollment as well as for the number of available servicing National Provider Identifiers in the Other Services File.

If using the DQ Atlas for data quality assessment, researchers should assess the detailed methodology for how each data quality measure was developed to determine whether it is aligned with their study objectives. In general, researchers should not solely rely on the DQ Atlas for assessing data quality in TAF. For instance, researchers may determine that the benchmarks used in the DQ Atlas to classify level of concern are overly generous or restrictive. For example, the data quality threshold for low concern for the race and ethnicity field in the DQ Atlas is defined as 10% or less allowable missingness and zero race and ethnicity categories where TAF differs from the American Community Survey by more than 10%. For a study focused on a specific racial or ethnic group enrolled in Medicaid, this level of specificity may be insufficient. Further, the DQ Atlas was developed on a 100% TAF sample, and data quality may differ for specific subpopulations, such as children, pregnant persons, individuals dually eligible for Medicare and Medicaid, or subgroups with specific diagnoses. Researchers may also be concerned about the data quality of elements that are not included in the DQ Atlas. In these cases, researchers should develop and report on their own external benchmarks. For example, one published study benchmarked Medicaid-paid births in the TAF to the US Centers for Disease Control and Prevention’s natality data files, including states in which the discrepancy was less than 20%.^19^ Other potential benchmarks exist, including CMS-64 form reports for spending and the Medicaid State Drug Utilization Data for prescription drug use.^20,21^ In cases where no external benchmarks exist, researchers may consider identifying and excluding outlier states; reporting should make clear how outliers were identified and the thresholds used for exclusion.

In all cases, it is recommended that researchers consider including an appendix table that summarizes key data quality measures for states both included and excluded from their primary analysis. Researchers should also consider conducting and reporting the results of sensitivity analyses in which they vary their data quality thresholds for state inclusion.

### Special Considerations

There are 2 additional issues that researchers should address in reporting on TAF-derived research.

First, analyses of spending in the TAF data can be particularly challenging given that payments on managed care encounters reflecting reimbursement from plans to health care facilities and professionals are redacted. As a result, researchers should again be careful to report whether their analysis included both fee-for-service and managed care encounters and the criteria used to make any exclusions. Researchers should also make clear what types of claims records were used to measure spending; for example, analyses of service-specific spending (eg, primary care physician visits) may be restricted to fee-for-service and managed care encounter records, while analyses of total state-level spending could be restricted to capitation and supplemental payments from the state to plans as well as health care facilities and professionals.

Second, TAF data are available for only a subset of states in 2014 and 2015; researchers conducting national longitudinal analyses extending prior to 2016 will therefore have to combine the MAX files and TAF. Given changes in data structure and quality in the transition from the MAX files to TAF, researchers should (1) report whether their analysis included MAX data and (2) assess trends in key outcomes, covariates, and population characteristics over the MAX to TAF transition in each state to ensure any observed changes over time are not artifacts of the data transition.

## Discussion

TAF data have much promise to catalyze research on the Medicaid program, but variation in data quality across states and time can introduce nontrivial bias and measurement error. The TAF Analysis Reporting Checklist represents a consensus effort to identify items researchers should report in TAF-based studies to allow editors, peer reviewers, and readers to fully assess how TAF data quality was addressed. This reporting is a key step in safeguarding the quality of research used to inform Medicaid policy.

The TAF Analysis Reporting Checklist is meant to complement–not substitute for–existing checklists, such as the STROBE and RECORD reporting guidelines. For example, the STROBE checklist encourages researchers to include a flowchart summarizing how inclusion and exclusion criteria affect the sample size,^8^ and the RECORD checklist asks authors to provide “a complete list of codes and algorithms used to classify exposures, outcomes, confounders, and effect modifiers.”^9^ Both of these recommendations are particularly important for TAF-derived research. Notably, the items included in the TAF Analysis Reporting Checklist are recommendations and may not apply to all studies.

### Limitations

The TAF Analysis Reporting Checklist is the result of a structured process to identify and document best practices to optimize the analysis and reporting of studies using TAF data. That said, the checklist was primarily developed within the MDLN, which does not include research teams from for-profit entities or across all types of institutions using the claims data. However, a draft of the checklist was presented publicly, and comments were welcomed from MDLN members and nonmembers. The TAF Analysis Reporting Checklist also was not validated empirically, but this step is not specifically recommended by the Guidance for Developers of Health Research Reporting Guidelines.^7^ Additionally, the checklist was developed specifically for application to the TAF data; it generalizes imperfectly to research using the MAX data and may not fully generalize to research using other Medicaid claims datasets, including claims data obtained directly from state Medicaid programs or other sources, such as private data vendors.

## Conclusions

Recent work indicates that TAF data quality represents a significant improvement relative to quality in legacy claims datasets, but quality is still not sufficient to enable most analyses of data from all states and territories for a given year.^16,22,23^ Variation in quality across data elements, states, and time means that researchers using TAF should thoughtfully assess data quality to make inclusion and exclusion decisions; researchers should communicate the potential effects of these decisions on bias and generalizability of findings alongside detail on the sensitivity of their results to alternative data quality thresholds. Transparent reporting on these decisions is critical for consumers of TAF-derived research to correctly interpret the conclusions of the research to inform evidence-based policies.

## Supplementary Material

Supplemental Online Content **eTable.** The T-MSIS Analytic Files (TAF) Analysis Reporting Checklist for Supplementary Material

## Figures and Tables

**Table.** The T-MSIS Analytic Files (TAF) Analysis Reporting Checklist and Reporting Examples^a^

Category	Description	Example text
**Data**		
Files, years, release versions, and data extract	• Indicate which TAF files were used in the analysis (eg, Demographic and Eligibility File, Inpatient File, Other Services File) • Indicate which years of TAF data were included in the analysis • Indicate which file versions were included in the analysis (eg, preliminary, release 1, release 2) • Indicate whether the study drew from 100% TAF files or prespecified extracts	• “Data for this study came from the TAF Other Services and Annual Provider Files for 2017 (Release 2), 2018 (Release 2), and 2019 (Release 1).” • “For this analysis, we used Release 1 of the Inpatient File for data years 2019 and 2020.” • “Please see the eTable in the Supplement for a summary of which files and TAF release versions were used by year.” • “We generated our study cohort from a 100% TAF sample for 2017-2018 Release 2.” • “We derived our study cohort from a 2016 Release 1 20% sample of Medicaid beneficiaries between the ages of 19 and 44 years in Texas, California, and New York.”
**Analytic sample**		
Eligibility criteria	• If applicable, describe what eligibility category codes were used to identify the study sample and whether they were used in combination with any other variables (eg, age, receipt of specific medical services)	• “We limited our analysis to individuals eligible under the Affordable Care Act’s Medicaid expansion as identified by an eligibility group code (ELGBLTY_GRP_CD) of 72, 73, 74, 75 observed latest in the year.”
Enrollment span	• If applicable, indicate the minimum period of enrollment required for an enrollee to be included in the study sample and how the enrollment period was defined	• “We limited our analysis to individuals with at least 6 months of continuous enrollment.” • “In our analysis, we required enrollees to be enrolled for a minimum of 12 months during the 3-year study period, though these months were not required to be consecutive.”
Scope of benefits	• If applicable, indicate whether the analysis included enrollees with full-scope, comprehensive, or restricted benefits	• “Our sample was limited to individuals with full-scope or comprehensive benefits based on their benefit status for the plurality of months they were enrolled in Medicaid in the year.” • “We excluded any individuals who were receiving only restricted Medicaid benefits during any month of our study period.”
Encounter data	• Indicate whether the analysis excluded either fee-for-service or managed care enrollees; if managed care enrollees were excluded, define the criteria used to do so • Indicate which types of claims records (eg, fee-for-service claims, service tracking claims, capitation payments; see variable CLM_TYPE_CD) were included in the analysis	• “Enrollees participating in comprehensive managed care plans for more than 6 months in the year were included in the analysis.” • “We limited our analysis to fee-for-service and managed care encounters.”
Dual eligibility	• Describe whether individuals dually enrolled in Medicare and Medicaid were included in or excluded from the study sample and, if applicable, how dual eligibility was defined	• “We excluded any individual with dual eligibility for both Medicaid and Medicare in any month of the year from our analysis.” • “We excluded those with partial or full dual eligibility based on the monthly eligibility status observed latest in the year. Individuals with unknown dual eligibility status were not excluded.”
**State and territory exclusions**		
Criteria	• Indicate which states and/or territories were included (or excluded) from the analysis on the basis of data quality concerns • Indicate the criteria by which state exclusions were made, including measures, data sources, and thresholds	• “We excluded from our analysis any state identified as high concern or unusable in the Data Quality Atlas for total Medicaid and Children’s Health Insurance Program enrollment. We additionally excluded any state where greater than 10% of servicing National Provider Identifiers were missing across noninstitutional fee-for-service and managed care records.” • “We excluded all states with live birth volumes that differed more than 20% from the number of Medicaid-financed births recorded in the US Centers for Disease Control and Prevention Natality data.” • “We restricted to states in which the missingness rate for race and ethnicity was less than 10% and non-Hispanic Black and non-Hispanic White race values did not differ from American Community Survey data by more than 10 percentage points.”
State variation table	• Consider including a state-level table (which may appear in an appendix) summarizing the number of observations, means, medians, and missingness for key study measures	• “Please see the eTable in the Supplement for a summary of the sample size and mean rates of emergency department visits for all states included in the analysis and for selected states that were excluded from the study.”
**Special considerations**		
Spending	• Indicate which types of claims records (eg, fee-for-service claims, service tracking claims, capitation payments; see variable CLM_TYPE_CD) were included to measure spending • If including service-specific spending for managed care encounters, indicate how spending was imputed (payments from plans to health care facilities and professionals on encounter records are generally redacted)	• “We created a measure of total spending per enrollee inclusive of fee-for-service claims, capitation payments, supplemental payments, and non–claims-based financial transactions.” • “We calculated total spending on primary care–specific services across fee-for-service and managed care encounters. We imputed spending on managed care encounters based on the median fee-for-service value for that service in the state.”
Using TAF with predecessor MAX data	• Indicate if the analysis included data from the MAX files and, if so, for what years and which states • If applicable, include an exhibit examining trends in key measures by state over time and particularly during any transition from MAX to TAF	• “Primary data came from the Medicaid Analytic eXtract for 2014-2015 and the T-MSIS Analytic Files for 2016-2018.” • “Please see Appendix Figure A1, which examines mean rates of emergency department visits during our study period across all states. States were excluded from the analysis if they had rate changes greater than 25% during the transition from MAX to TAF.”

Abbreviation: MAX, Medicaid Analytic eXtract.

a The TAF Analysis Reporting Checklist includes reporting recommendations for TAF-based analyses. Authors of TAF-based research should indicate whether they followed the checklist in their manuscripts; an eTable in the supplementary material may be helpful to indicate whether and how the authors complied with each recommendation. Checklist items are recommendations, not prescriptions, and can be adapted to different study designs. Not all checklist items will apply to all studies. The TAF Analysis Reporting Checklist is not meant to be a substitute for broader reporting checklists (eg, the STROBE reporting guideline for observational research or the RECORD reporting guideline for research using routinely collected health data). Rather, it is meant to complement those resources with guidance on TAF-specific methodological considerations. See the eTable in the Supplement for a version of the checklist to include in study appendices.

## References

[1] Mykyta L, Keisler-Starkey K, Bunch L. More children were covered by Medicaid and CHIP in 2021. Accessed January 31, 2025. https://www.census.gov/library/stories/2022/09/uninsured-rate-of-children-declines.html

[2] Medicaid and CHIP Payment and Access Commission. Estimates of Medicaid nursing facility payments relative to costs. Accessed January 31, 2025. https://www.macpac.gov/wp-content/uploads/2023/01/Estimates-of-Medicaid-Nursing-Facility-Payments-Relative-to-Costs-1-6-23.pdf

[3] US Centers for Medicare & Medicaid Services. 2024 Medicaid & CHIP beneficiaries at a glance: maternal health. Accessed January 31, 2025. https://www.medicaid.gov/medicaid/benefits/downloads/2024-maternal-health-at-a-glance.pdf

[4] US Centers for Medicare & Medicaid Services. NHE fact sheet. Accessed January 31, 2025. https://www.cms.gov/data-research/statistics-trends-and-reports/national-health-expenditure-data/nhe-fact-sheet

[5] Turner L, Shamseer L, Altman DG, Schulz KF, Moher D. Does use of the CONSORT Statement impact the completeness of reporting of randomised controlled trials published in medical journals? a Cochrane review. Syst Rev. 2012;1(1):60. doi:10.1186/2046-4053-1-60 23194585 PMC3564748

[6] Pouwels KB, Widyakusuma NN, Groenwold RHH, Hak E. Quality of reporting of confounding remained suboptimal after the STROBE guideline. J Clin Epidemiol. 2016;69:217–224. doi:10.1016/j.jclinepi.2015.08.009 26327488

[7] Moher D, Schulz KF, Simera I, Altman DG. Guidance for developers of health research reporting guidelines. PLoS Med. 2010;7(2):e1000217. doi:10.1371/journal.pmed.1000217 20169112 PMC2821895

[8] von Elm E, Altman DG, Egger M, Pocock SJ, Gøtzsche PC, Vandenbroucke JP; STROBE Initiative. The Strengthening the Reporting of Observational Studies in Epidemiology (STROBE) statement: guidelines for reporting observational studies. Ann Intern Med. 2007;147(8):573–577. doi:10.7326/0003-4819-147-8-200710160-00010 17938396

[9] Benchimol EI, Smeeth L, Guttmann A, RECORD Working Committee. The Reporting of Studies Conducted Using Observational Routinely-Collected Health Data (RECORD) statement. PLoS Med. 2015;12(10):e1001885. doi:10.1371/journal.pmed.1001885 26440803 PMC4595218

[10] Auty SG, Daw JR, Admon LK, Gordon SH. Comparing approaches to identify live births using the Transformed Medicaid Statistical Information System. Health Serv Res. 2024;59(1):e14233. doi:10.1111/1475-6773.14233 37771156 PMC10771902

[11] Sommers BD. Loss of health insurance among non-elderly adults in Medicaid. J Gen Intern Med. 2009;24(1):1–7. doi:10.1007/s11606-008-0792-9 18810555 PMC2607511

[12] Daw JR, Hatfield LA, Swartz K, Sommers BD. Women in the United States experience high rates of coverage ‘churn’ in months before and after childbirth. Health Aff (Millwood). 2017;36(4):598–606. doi:10.1377/hlthaff.2016.1241 28373324

[13] US Centers for Medicare & Medicaid Services. Identifying beneficiaries with full-scope, comprehensive, and limited benefits in the TAF. Accessed May 7, 2025. https://resdac.org/sites/datadocumentation.resdac.org/files/2021-01/4151_Scope_of_Benefits.pdf

[14] Becerra X. Expanded Report to Congress: non-emergency medical transportation in Medicaid, 2018-2021. Accessed May 7, 2025. https://www.medicaid.gov/medicaid/benefits/downloads/nemt-rtc-2018-2021.pdf

[15] KFF. Total Medicaid MCO enrollment. Accessed January 31, 2025. https://www.kff.org/other/state-indicator/total-medicaid-mco-enrollment/

[16] Samples H, Lloyd K, Ryali R, Completeness and quality of comprehensive managed care data compared with fee-for-service data in national Medicaid claims from 2001 to 2019. Health Serv Res. 2025;60(3):e14429. doi:10.1111/1475-6773.14429 39748217 PMC12120513

[17] Lloyd K, Rege S, Crystal S, Olfson M, Horton DB, Samples H. Completeness and quality of data for children in Medicaid comprehensive managed care compared to fee-for-service, 2001–2019. Health Serv Res. 2025;e14651. doi:10.1111/1475-6773.14651 40464879 PMC12636249

[18] Medicaid.gov. DQ Atlas. Accessed May 7, 2025. https://www.medicaid.gov/dq-atlas/welcome

[19] Admon LK, Auty SG, Daw JR, State variation in severe maternal morbidity among individuals with Medicaid insurance. Obstet Gynecol. 2023;141(5):877–885. doi:10.1097/AOG.0000000000005144 37023459 PMC10281794

[20] Medicaid.gov. Expenditure reports from MBES/CBES. Accessed June 17, 2025. https://www.medicaid.gov/medicaid/financial-management/state-expenditure-reporting-for-medicaid-chip/expenditure-reports-mbescbes

[21] Medicaid.gov. State drug utilization data. Accessed June 17, 2025. https://www.medicaid.gov/medicaid/prescription-drugs/state-drug-utilization-data

[22] Nguyen JK, Sanghavi P. A national assessment of legacy versus new generation Medicaid data. Health Serv Res. 2022;57(4):944–956. doi:10.1111/1475-6773.13937 35043402 PMC9264472

[23] Wang S, Qi M, Konetzka RT. Home- and community-based care in the new generation of Medicaid administrative data. Health Serv Outcomes Res Methodol. 2025;25(1):29–41. doi:10.1007/s10742-024-00325-6 39958809 PMC11821789

